# Age-Friendly Community Practice: Understanding the Role of Community Events

**DOI:** 10.1093/geroni/igab046.2138

**Published:** 2021-12-17

**Authors:** Natalie Pope, Emily Greenfield, Althea Pestine-Stevens, Clara Scher

**Affiliations:** 1 Rutgers Social Work, New Brunswick, New Jersey, United States; 2 Rutgers, The State University of New Jersey, New Brunswick, New Jersey, United States

## Abstract

To advance an emerging empirical knowledge base for age-friendly community initiatives (AFCIs), we conducted a qualitative descriptive study to explore one manifestation of age-friendly practice: community events (CEs). We aimed to illuminate how AFCI core teams describe CEs as part of their practice and how they perceive the value of CEs for age-friendly progress. Using inductive coding, we analyzed data from semi-structured interviews (n=24) with eight core teams across three time points spanning the early to mid-implementation phases of the AFCIs. Two predominant themes emerged. First, CEs were described as important for working toward age-friendly goals concerning older residents’ social participation and inclusion. Second, core teams described the longer-term strategic value of CEs, such as building interorganizational partnerships; providing deeper insight on aging in community; and fostering older adults’ leadership as part of the initiative. We discuss implications for advancing transdisciplinary program theory to guide more sustainable and effective AFCIs.

